# How high must lactate be to predict an adverse outcome?

**DOI:** 10.1186/cc9930

**Published:** 2011-03-11

**Authors:** A Reintam Blaser, J Starkopf

**Affiliations:** 1 University of Tartu, Estonia

## Introduction

We aimed to clarify the associations between lactate levels and ICU mortality and their changes over 6 years in one ICU.

## Methods

All patients admitted to the general ICU of university hospital from 2005 to 2010 were studied. Highest lactate on admission day in the ICU was documented.

## Results

**Figure 1:**
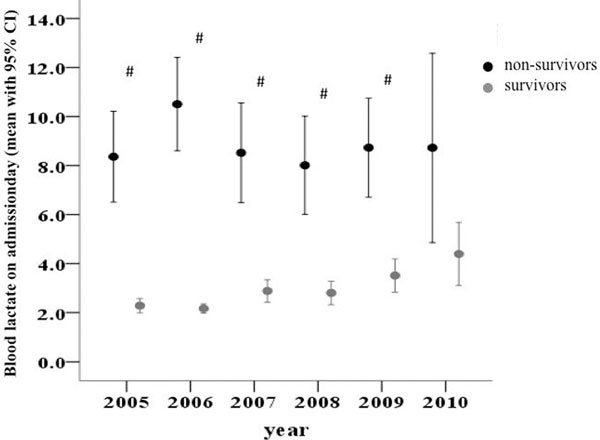
**Lactate levels of survivors versus nonsurvivors over the years**. ^#^Difference between survivors and nonsurvivors, *P *< 0.005.

**Figure 2:**
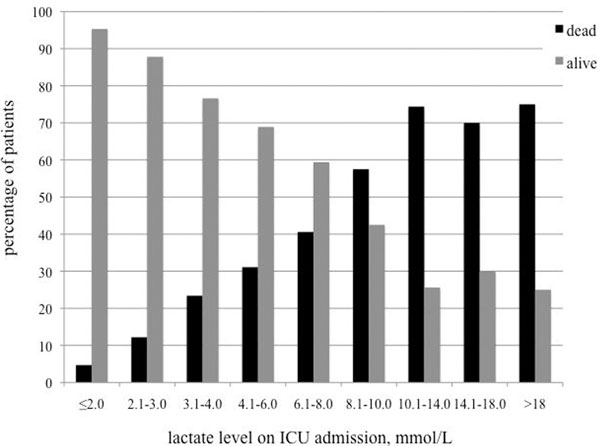
**ICU survival rates according to lactate levels**.

In total, 1,830 patients were treated, 417 were excluded due to incomplete data and 1,413 patients were included in the study. Survivors had a mean blood lactate level of 2.8 ± 3.3 versus 8.9 ± 7.2 mmol/l in nonsurvivors (*P *< 0.001). The lactate levels of survivors versus nonsurvivors over the years are presented in Figure 1. The survival in different lactate groups is presented in Figure 2.

## Conclusions

There is a linear correlation between blood lactate levels and ICU mortality. A considerable amount of patients with very high lactate levels survive the ICU. There is no certain lactate level that may reliably predict an adverse outcome.

